# Smartphone-Enabled versus Conventional Otoscopy in Detecting Middle Ear Disease: A Meta-Analysis

**DOI:** 10.3390/diagnostics12040972

**Published:** 2022-04-13

**Authors:** Chih-Hao Chen, Chii-Yuan Huang, Hsiu-Lien Cheng, Heng-Yu Haley Lin, Yuan-Chia Chu, Chun-Yu Chang, Ying-Hui Lai, Mao-Che Wang, Yen-Fu Cheng

**Affiliations:** 1 Department of Otolaryngology-Head and Neck Surgery, Taipei Veterans General Hospital, Taipei 112, Taiwan; michaelchen808@gmail.com (C.-H.C.); dopod0635@gmail.com (C.-Y.H.); hlcheng@vghtpe.gov.tw (H.-L.C.); haleylin0222@gmail.com (H.-Y.H.L.); 2 Faculty of Medicine, National Yang Ming Chiao Tung University, Taipei 112, Taiwan; 3 Department of Biomedical Engineering, National Yang Ming Chiao Tung University, Taipei 112, Taiwan; yh.lai@nycu.edu.tw; 4 Department of Information Management, Taipei Veterans General Hospital, Taipei 112, Taiwan; xd.yuanchia@gmail.com; 5 Big Data Center, Taipei Veterans General Hospital, Taipei 112, Taiwan; 6 Department of Information Management, National Taipei University of Nursing and Health Sciences, Taipei 112, Taiwan; 7 Department of Anesthesiology, Taipei Tzu Chi Hospital, Buddhist Tzu Chi Medical Foundation, New Taipei City 231, Taiwan; paulchang1231@gmail.com; 8 Medical Device Innovation & Translation Center, National Yang Ming Chiao Tung University, Taipei 112, Taiwan; 9 Institute of Hospital and Health Care Administration, National Yang Ming Chiao Tung University, Taipei 112, Taiwan; 10 Department of Medical Research, Taipei Veterans General Hospital, Taipei 112, Taiwan; 11 Institute of Brain Science, National Yang Ming Chiao Tung University, Taipei 112, Taiwan

**Keywords:** smartphone-enabled otoscopy, conventional otoscopy, middle ear disease

## Abstract

Traditional otoscopy has some limitations, including poor visualization and inadequate time for evaluation in suboptimal environments. Smartphone-enabled otoscopy may improve examination quality and serve as a potential diagnostic tool for middle ear diseases using a telemedicine approach. The main objectives are to compare the correctness of smartphone-enabled otoscopy and traditional otoscopy and to evaluate the diagnostic confidence of the examiner via meta-analysis. From inception through 20 January 2022, the Cochrane Library, PubMed, EMBASE, Web of Science, and Scopus databases were searched. Studies comparing smartphone-enabled otoscopy with traditional otoscopy regarding the outcome of interest were eligible. The relative risk (RR) for the rate of correctness in diagnosing ear conditions and the standardized mean difference (SMD) in diagnostic confidence were extracted. Sensitivity analysis and trial sequential analyses (TSAs) were conducted to further examine the pooled results. Study quality was evaluated by using the revised Cochrane risk of bias tool 2. Consequently, a total of 1840 examinees were divided into the smartphone-enabled otoscopy group and the traditional otoscopy group. Overall, the pooled result showed that smartphone-enabled otoscopy was associated with higher correctness than traditional otoscopy (RR, 1.26; 95% CI, 1.06 to 1.51; *p* = 0.01; *I*^2^ = 70.0%). Consistently significant associations were also observed in the analysis after excluding the simulation study (RR, 1.10; 95% CI, 1.00 to 1.21; *p* = 0.04; *I^2^* = 0%) and normal ear conditions (RR, 1.18; 95% CI, 1.01 to 1.40; *p* = 0.04; *I^2^* = 65.0%). For the confidence of examiners using both otoscopy methods, the pooled result was nonsignificant between the smartphone-enabled otoscopy and traditional otoscopy groups (SMD, 0.08; 95% CI, -0.24 to 0.40; *p* = 0.61; *I*^2^ = 16.3%). In conclusion, smartphone-enabled otoscopy was associated with a higher rate of correctness in the detection of middle ear diseases, and in patients with otologic complaints, the use of smartphone-enabled otoscopy may be considered. More large-scale studies should be performed to consolidate the results.

## 1. Introduction

Otoscopy is an important and essential tool for the diagnosis of middle ear diseases. More often than not, diagnosis of middle ear disease is made from the direct observation of the appearance of the tympanic membrane and the middle ear cavity, in conjunction with clinical manifestations [1,2]. However, traditional otoscopy has some limitations, including the requirement of experienced specialists, such as otolaryngologists or pediatricians, to perform immediate examination and interpretation of the results [3,4,5], and various hindrances associated with the clinical setting, such as poor visualization caused by a narrowed ear canal or insufficient examination time owing to the discomfort of the patient [1,6,7,8]. These shortcomings are more apparent in certain suboptimal environments, such as emergency departments [7,9]. As a result, clinicians are likely to evaluate these diseases inappropriately [10].

Video-assisted otoscopy records images of lesions observed with the otoscope through an auxiliary imaging system, which substantially improves the shortcomings associated with traditional otoscopy [8,11,12,13]. However, the costs of such imaging systems hinder their clinical application [8,14]. The advancement of technology and the rapid development of various medical-related applications that are compatible with smartphones have facilitated the use of smartphone imaging systems and monitoring systems [15,16,17,18,19,20,21,22,23,24,25]. Additionally, compared with the costs of traditional imaging systems, the total cost of the hardware and software needed for smartphone-enabled otoscopy is often much lower, which further promotes the use of smartphone-enabled otoscopy [18,19].

Smartphones in modern society are well equipped, and it is relatively easy for clinicians to become familiar with the use of smartphone-enabled otoscopy. In the coronavirus disease 2019 (COVID-19) post-epidemic era, the importance of telemedicine has been emphasized and valued [26,27,28,29,30,31,32,33,34]. Preventing unnecessary exposure through remote telemedicine has become an important issue in this era. The characteristics of smartphone-enabled otoscopy make it relatively easy to use remotely, allowing patients and physicians to discuss medical concerns digitally and to further assist in the diagnosis and management of diseases [17]. Under these circumstances, the cost-effectiveness of smartphone-enabled otoscopy and the inherent advantage of telemedicine greatly potentializes smartphone-enabled otoscopy as a modern first-line tool for the diagnosis of middle ear diseases.

Recently, studies have verified the efficacy and benefit of smartphone-enabled otoscopy [9,35]. However, the method remains controversial compared to traditional otoscopy, which is still the mainstream middle ear examination method. Additionally, studies have also suggested that diagnostic confidence is favorable for examiners when using smartphone-enabled otoscopy; however, the issue remains unclear [36]. Accordingly, the present study aims to provide comprehensive evidence by systematically reviewing and meta-analyzing the current literature and comparing the correctness and user confidence between smartphone-enabled otoscopy and traditional otoscopy.

## 2. Materials and Methods

### 2.1. Study Design

This systematic review and meta-analysis followed the preferred reporting items for systematic reviews and meta-analyses (PRISMA) guidelines [13,37]. The patient data used in this systematic review and meta-analysis were de-identified, and approval from the institutional review board or the ethical committee and informed consent was not required given that the institutional review board (IRB) of Taipei Veterans General Hospital states that data from the public environment and from de-identified information do not need an audit by the IRB or the ethical committee or informed consent. Additionally, the International Prospective Register of Systematic Reviews (PROSPERO) was registered [CRD42021262227].

### 2.2. Search Strategy

From inception through 20 January 2022, databases, including the Cochrane Library, PubMed, EMBASE, Web of Science, and Scopus, were searched. We used a combination of Medical Subject Headings (MeSH) and text words to create three citation subsets: one included studies on smartphone-enabled examination (“Smartphones”, “Cellular Phone”, “Mobile Phone”), one included studies on traditional examination (“Traditional” OR “Conventional” OR “Standard”) and one included otoscopy as the examination method (“Otoscopes” OR “Otoscope” OR “Otoscopy” OR “Otoscopies”). The detailed search strategy is shown in Supplementary (Table S1).

### 2.3. Eligibility Criteria and Study Inclusion

Included studies were selected according to the following criteria: the study compared smartphone-enabled otoscopy with traditional otoscopy regarding the outcome of interest (i.e., rate of correctness), and the study provided adequate information to quantify the effect estimates for meta-analysis. The titles, abstracts and keywords of identified records were screened. The full texts of eligible records were then reviewed. After review by two authors (C.-H. Chen and C.-Y. Chang), the effect estimates of interest were extracted. Primary data were analyzed to evaluate the rate of correctness in identifying ear conditions, including normal conditions or diseased conditions, in both the smartphone group and traditional group. Other outcomes, including confidence scoring, were also extracted for meta-analysis.

### 2.4. Data Management

The data were randomly allocated into two examination arms in the present study; in one arm, the examiner and examinee performed smartphone-enabled otoscopy, and in the other arm, the examiner and examinee performed traditional otoscopy. The diagnosis received by the examinee was either verified by an experienced otolaryngologist or pediatrician or directly designed on the dummy simulation. The risk ratio (RR) for correctness in diagnosing an ear condition and the standardized mean difference (SMD) in diagnosis confidence between the groups were calculated. 

### 2.5. Risk of Bias Assessment

The revised Cochrane risk of bias tool 2 was applied to evaluate the methodological quality of the included studies [38].

### 2.6. Statistical Analysis

**Figure 1:**
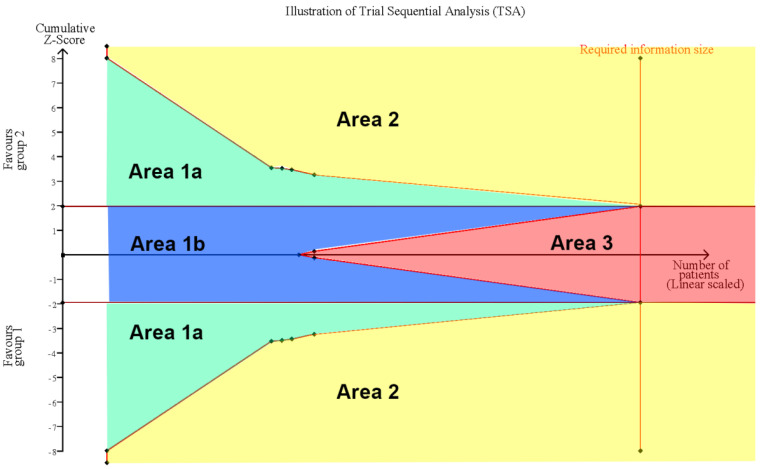
Illustration of Trial Sequential Analysis (TSA). Area 1 between sequential monitoring boundary and inner wedge indicated the inconclusive result which may suffer from false positive (Area 1a) or false negative (Area 1b), more sample size is required for further consolidate conclusion. Area 2 demonstrate conclusive result of significant effect of experimental group or control group, while Area 3 indicate the conclusive result of non-significance between experimental group and control group.

The random-effects model was used for the effect size calculation under the assumption that a second source of error other than sampling error existed. Statistical heterogeneity was assessed by the Cochran Q test and the *I*^2^ statistic. Heterogeneity was regarded as low, moderate, and high at *I*^2^ values of <50%, 50–74%, and ≥75%, respectively. In addition, sensitivity analyses were performed by (1) excluding normal conditions within the ear of the examinee and (2) excluding examination comparisons via dummy simulations. All the calculations for the meta-analysis were performed in R studio with the metaphor package. Additionally, trial sequential analysis (TSA) was performed to evaluate whether the result was subject to type I or type II errors caused by a lack of data or power by using TSA software, version 0.9.5.10 Beta [39,40]. In the TSA, the conventional significance boundary represents the general confidence interval (CI) we used in the meta-analysis. A meta-analysis represents a similar process of sampling tests, and type I or type II error may occur. Under these circumstances, an adjusted CI according to the sample size is needed; that is, when the sample size is small, a large CI would be expected, since the standard error would be large. The CI would gradually decrease as the sample size increases, and these adjusted CIs could be connected and form the sequential monitoring boundary. The required information size (RIS) was calculated under the setting of the current difference between the control group and the experimental group. We created an illustration for the interpretation of the TSA results (Figure 1). In this illustration, Area 1, between the sequential monitoring boundary and inner wedge indicated an inconclusive result that may suffer from false positives or false negatives, and a larger sample size is required to further consolidate the conclusion. Area 2 demonstrates the conclusive result of a significant effect of the experimental group or control group, while Area 3 indicates the conclusive result of nonsignificant differences between the experimental group and the control group. The conventional significance boundary in TSA analysis was −1.96 to 1.96, and the sequential monitoring boundary varied by analysis. The models for all outcomes were assessed considering an alpha value of 0.05 and a power of 80%.

## 3. Results

### 3.1. Study Identification and Selection

**Figure 2:**
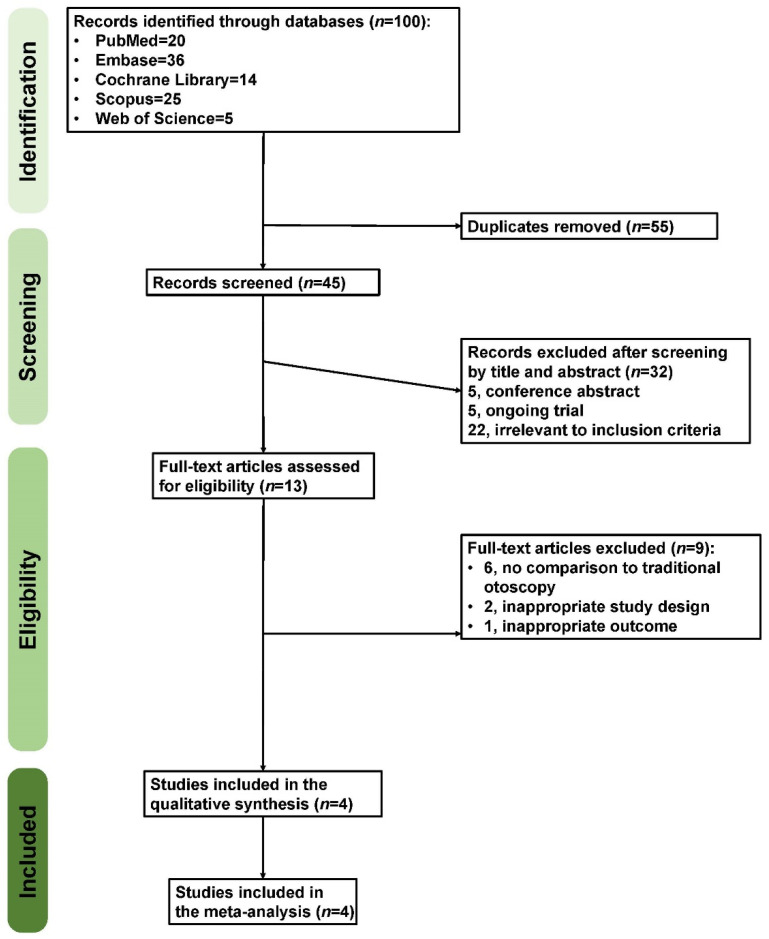
The Preferred Reporting Items for Systematic Reviews and Meta-Analyses (PRISMA) flow diagram.

The present study identified 100 records in the preliminary search. After removing duplicates and screening titles and abstracts, 13 studies eventually underwent full-text review. Nine studies were excluded due to a lack of comparison to traditional otoscopy, an inappropriate study design, or an inappropriate outcome. As a result, four eligible randomized controlled trials (RCTs) were included [11,41,42,43], as presented in the PRISMA flow chart (Figure 2). 

### 3.2. Study Characteristics

A total of 1840 examinees were divided into the smartphone-enabled otoscopy group and the traditional otoscopy group. Three studies compared pediatric patients with acute otitis media (AOM) who were evaluated in the emergency room, while one study used a dummy simulation for the comparison of both groups [43]. The study that used a dummy simulation compared four independent settings. As a result, these groups were included in the meta-analysis [43]. All the studies used smartphone-enabled otoscopy with an iOS system [11,41,42,43]. In two studies, a comparison of diagnostic confidence associated with smartphone-enabled otoscopy and traditional otoscopy was also reported [11,43]. Two studies were composed of residents in the emergency department [41,42], one study consisted of medical students [43], and another study was comprised of both residents and medical students [11]. Detailed information is presented in Table 1.

### 3.3. Risk of Bias Assessment

The risk of bias was assessed in each of the included studies. We present the detailed assessment in Supplementary (Figures S1 and S2). All the studies clearly presented the randomization procedure. However, none of the studies could blind the examiners and examinees to the type of examination. Additionally, the outcome assessors were aware of the type of examinations received by the study participants. None of the four studies reported whether the measurement or ascertainment of the outcome differed between the intervention groups. Regarding all the above, there was some concern regarding the risk of bias.

### 3.4. Overall Comparison between Smartphone-Enabled Otoscopy and Traditional Otoscopy

**Figure 3:**
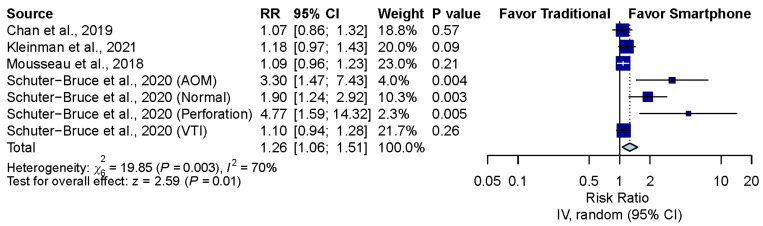
Overall comparison between smartphone-enabled otoscopy and traditional otoscopy [11,41,42,43].

Four studies, including seven groups, compared the correctness of diagnosis between the smartphone-enabled otoscopy and traditional otoscopy groups [11,41,42,43]. Overall, the pooled result showed that smartphone-enabled otoscopy was associated with a higher rate of correctness (RR, 1.26; 95% CI, 1.06 to 1.51; *P* = 0.01; *I*^2^ = 70.0%) (Figure 3).

### 3.5. Sensitivity Analysis after Excluding the Simulation Study

**Figure 4:**
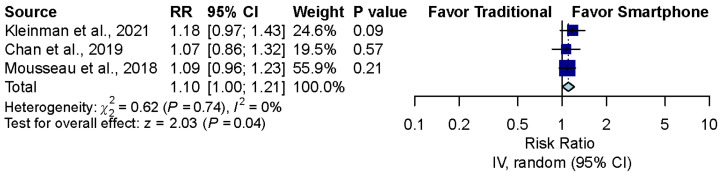
Sensitivity analysis after excluding the simulation study [11,41,42].

The pooled effect estimates of the three studies that assessed otoscopy on patients with acute otitis media (AOM) in the emergency department revealed that smartphone-enabled otoscopy was associated with a higher rate of correctness [11,41,42] (RR, 1.10; 95% CI, 1.00 to 1.21; *p* = 0.04; *I*^2^ = 0%) (Figure 4).

### 3.6. Sensitivity Analysis after Excluding Normal Ear Conditions

**Figure 5:**
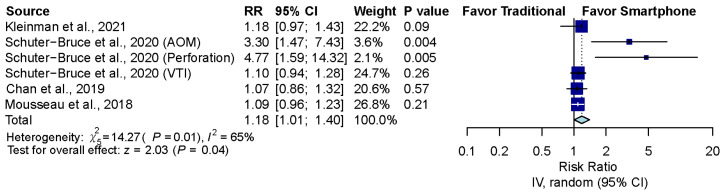
Sensitivity analysis after excluding normal ear conditions [11,41,42,43].

The pooled effect estimates of four studies which included six groups that assessed otoscopy on patients with abnormal ear conditions, including AOM, perforation and ventilation tube insertion (VTI), showed that smartphone-enabled otoscopy was generally associated with a higher rate of correctness in detecting abnormal ear conditions [11,41,42,43]. (RR, 1.18; 95% CI, 1.01 to 1.40; *p* = 0.04; *I*^2^ = 65.0%) (Figure 5).

### 3.7. Confidence Comparison between Smartphone-Enabled Otoscopy and Traditional Otoscopy

**Figure 6:**
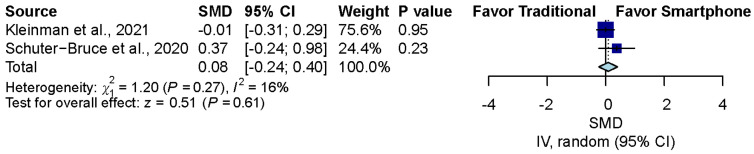
Confidence comparison between smartphone-enabled otoscopy and traditional otoscopy [11,43].

Considering the two studies that reported the confidence of examiners using both otoscopy methods [11,43], the pooled result was nonsignificant between the smartphone-enabled otoscopy and traditional otoscopy groups (SMD, 0.08; 95% CI, −0.24 to 0.40; *p =* 0.61; *I*^2^ = 16.3%) (Figure 6).

### 3.8. Influence Analysis and Trial Sequential Analysis

**Figure 7:**
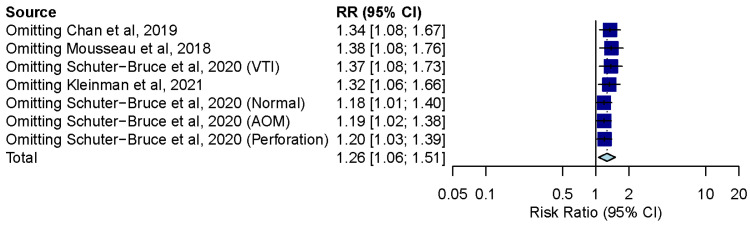
Influence analysis of the overall result of comparison between smartphone-enabled otoscopy and traditional otoscopy [11,41,42,43].

**Figure 8:**
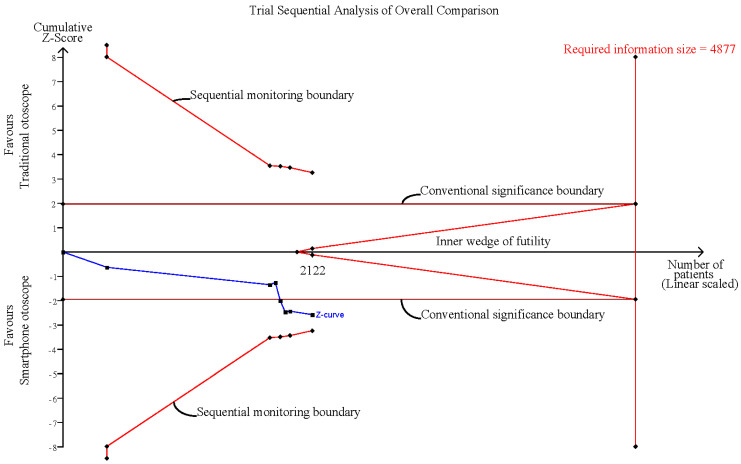
Trial sequential analysis (TSA) of overall comparison between smartphone-enabled otoscopy and traditional otoscopy.

**Figure 9:**
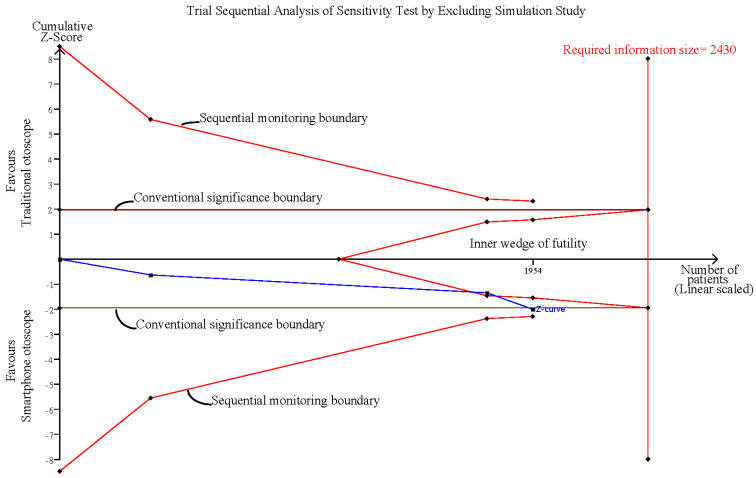
Trial sequential analysis (TSA) of sensitivity analysis by excluding the simulation study.

**Figure 10:**
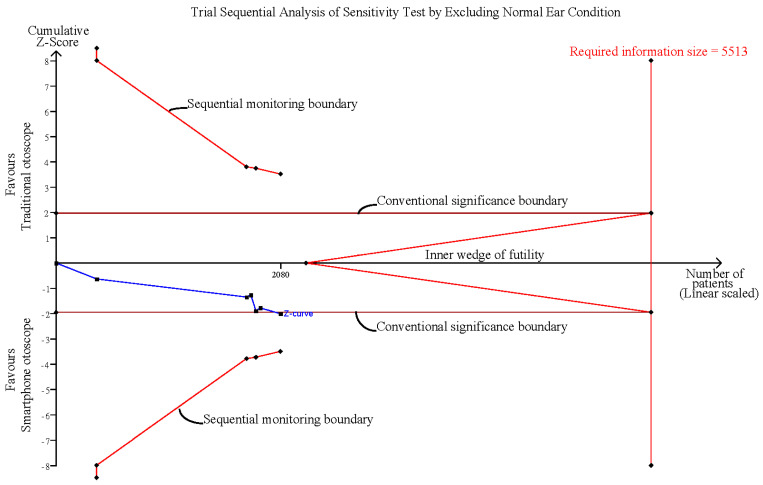
Trial sequential analysis (TSA) of sensitivity analysis by excluding normal ear conditions.

**Figure 11:**
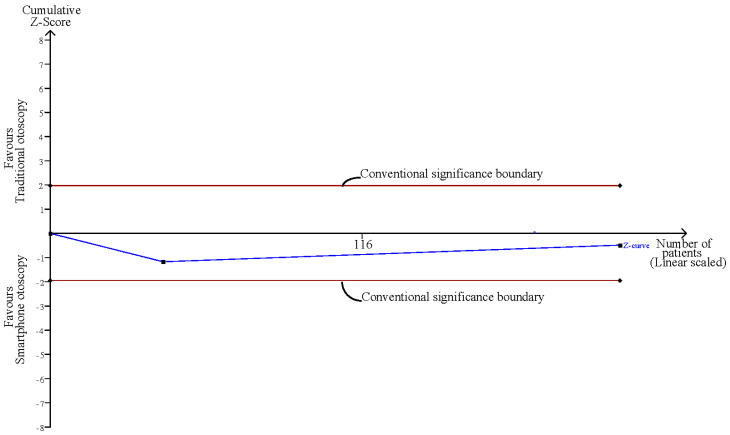
TSA of the confidence comparison between smartphone-enabled otoscopy and traditional otoscopy.

Influence analysis showed that after excluding each group one at a time, the pooled estimates remained within the 95% CI of the overall pooled results for these outcomes (Figure 7). TSAs of the meta-analysis results of the overall comparison, excluding simulation studies and normal ear conditions, showed that the cumulative z-curve exceeded the conventional significance boundary but not the sequential monitoring boundary. TSAs of the results of diagnostic confidence comparisons showed that the cumulative z-curve did not surpass either the conventional significance or sequential monitoring boundary (not renderable in the analysis, owning to the information size being too small). Additionally, none of the TSAs reached the suggested required information size (RIS) threshold (Figure 8, Figure 9, Figure 10 and Figure 11). As we mentioned in the methodology, these results suggest potential type I or type II errors with insufficient sample size.

## 4. Discussion

The paramount finding of the present study is that smartphone-enabled otoscopy was associated with a higher rate of correctness in various clinical settings. Further sensitivity analysis confirmed that smartphone-enabled otoscopy was consistently associated with a higher correctness rate in detecting abnormal tympanic lesions, and when performed in pediatric patients with AOM. To our knowledge, this is the first meta-analysis to provide direct evidence of the superiority of smartphone-enabled otoscopy compared to traditional otoscopy.

Otoscopy is an essential part of the physical examination that can identify critical pathologies of the ear canal and middle ear. However, consistency and confidence in performing otoscopy examinations have declined among medical personnel [44,45]. This insufficiency may be due in part to poor visualization associated with various clinical settings, poor otoscopy instruction and insufficient exposure to otolaryngology as part of the core clinical skills training in medical and primary care practitioner education [5,46,47]. Previous literature has reported that otoscopy has low sensitivity and specificity in the diagnosis of middle ear diseases, such as AOM [6,7,48]. Diagnosing external ear or middle ear diseases by the appearance and color of the ear canal or tympanic membrane is very challenging.

To overcome the disadvantages of traditional otoscopy, research on video-assisted otoscopy has increased in popularity in recent years [8,12,13]. Video-assisted otoscopy applications are commonly equipped with imaging systems, which can record images or even videos of the external ear or middle ear lesions on otoscopy. Clinicians can use the recorded images to carefully interpret findings, resulting in a more accurate diagnosis and fewer obstacles due to the aforementioned shortcomings of traditional otoscopy [8,15,49]. In addition to clinicians, this advantage also helps less experienced doctors, including interns and medical students, become familiar with diagnosing external ear and middle ear diseases and decreases the learning curve [8,14].

The biggest disadvantage of video-assisted otoscopy is that it usually needs to be connected to a relatively high-cost imaging system, which leads to considerable limitations in its clinical application [8,14,17,50]. Accordingly, smartphone-enabled otoscopy has prominent advantages. First, smartphones are widely used. People are becoming increasingly familiar with the use of smartphones, which helps promote the use of smartphone-enabled otoscopy. Second, with new technological advances, applications of smartphones with compatible imaging systems for otoscopy have become available, and applications are able to provide various functions according to different demands [15,16,17]. Third, the cost of the mobile phone and the imaging system is not as expensive as that of the traditional imaging system. According to previous research, smartphone-enabled otoscopy allows users to perform and digitally record middle ear examinations for as little as $30 [17,36]. These advantages make smartphone-enabled otoscopy more suitable for clinical applications, especially in environments that highly demand otoscopy examinations, such as emergency departments and local clinics. A previous report showed improved visualization of the tympanic membrane with smartphone-enabled otoscopy compared to traditional otoscopy [17,36]. Additionally, other studies have shown that in addition to eliminating some of the drawbacks of conventional otoscopy, smartphone-enabled otoscopy has certain benefits and efficacy, including the possibility of practicing remote telemedicine, improving both patient and clinician convenience in disease diagnosis and surveillance [9,19,51,52]. This feature is especially critical in the post-COVID-19 era as an alternative to visiting clinics or medical institutions to prevent COVID-19 exposure [17,28,29,53,54,55].

Another important finding of this study is that after excluding the simulation study involving medical students, analysis of the three examination studies involving mainly residents in the emergency department suggested that smartphone-enabled otoscopy was associated with a higher correctness rate than traditional otoscopy [11,41,42]. The studies subjected to sensitivity analysis included pediatric patients with AOM, and the examiners were not specialists. Previous studies have pointed out that the correctness of the otoscopy examination in the emergency department is significantly low [7,9,10,48,56]. There are several reasons for the poor performance of otoscopy. First, the majority of AOM patients in the emergency department are children. The external ear canal of children is relatively narrow and small, leading to poor visualization of the external ear and tympanic membrane [8,10]. Second, these children are less likely to cooperate with otoscopy examination, partly because of the discomfort associated with and the fear of examination, which makes it difficult for medical staff to observe the eardrum appropriately [10,41,56]. Furthermore, during an examination of pediatric patients, crying often leads to physiological congestion and redness of the eardrum, which may result in misdiagnosis as an inflamed eardrum [10]. Finally, in such a suboptimal environment, the provider is often forced to finish the examination quickly, resulting in insufficient time to evaluate the lesion and thus, creating potential misjudgment [10]. As AOM is mainly a clinical diagnosis, clinicians often overdiagnose AOM and inappropriately prescribe antibiotics when the criteria are not met in such an environment [6,7]. On the other hand, a missed diagnosis of AOM may evolve into complications or long-term sequelae, including acute mastoiditis, acute labyrinthitis or even chronic otitis media, if not diagnosed in a timely manner [57,58]. Considering the drawbacks and recent advances in infection control, some experts have suggested that the role of traditional otoscopy in diagnosing middle ear diseases, such as AOM, should be limited to prevent the unnecessary use of antibiotics and delayed diagnosis [10,49]. Improving correctness by performing smartphone-enabled otoscopy in suboptimal environments such as emergency rooms is promising, and we believe that smartphone-enabled otoscopy would outperform traditional otoscopy in other clinical and teaching scenarios. In our study, the pooled effect size showed a 26% increase in smartphones. After excluding the simulation study, the pooled effect size was reduced by 16% from the overall comparison. The possible reason is that the comparison between smartphone-based otoscopy and traditional otoscopy under the simulation scenario may sustain the eradication of confounding factors, including patient non-cooperativity, suboptimal environments and variations in disease conditions [1,2,3]. Under the simulation scenario, smartphone-enabled otoscopy and traditional otoscopy may show a greater difference (higher odds ratio). However, considering that these examinations are eventually applied in the clinical setting, a sensitivity test excluding the simulation study is essential. After the exclusion of the simulation test, the heterogeneity decreased, which may come from the consistency of the setting of the remaining three studies. Regarding the result that removal of the normal ear condition reduced the risk ratio by 8%, a previous study also mentioned the difference between smartphone-enabled otoscopy and traditional otoscopy in the identification of normal anatomy [36], and removal of the comparison detecting normal ear conditions may indeed decrease the overall pooled effect size. However, variation in the pooled effect size may also come from the random-effects model we used, which means that the true pooled effect size would not fix on the only value due to the between-study variation.

Finally, the diagnostic confidence comparison between the two groups was nonsignificant. A previous study indicated that correctness may not be correlated with confidence level [13]. Additionally, one of the included studies in this analysis included a simulation examination, which may omit the clinical information of the examinee. As diagnostic confidence may be partly derived from clinical information, insufficient information may cause examination providers to be more conservative and therefore attenuate the real confidence status [13,15]. Nevertheless, due to the small number of included studies and the TSA suggesting an inadequate sample size, the association between the otoscopy method and diagnostic confidence remains inconclusive, and we suggest further large-scale studies to validate the result.

### Limitations

First, the effect estimate in this article was calculated by comparing the correctness of two diagnostic methods in certain situations. Although the results indicated that smartphone-enabled otoscopy is more accurate in diagnosing middle ear diseases, we were unable to evaluate the full diagnostic performance of smartphone-enabled otoscopy. This requires the usage of gold standard examinations as reference tests (i.e., tympanometry) and additional clinical validation to compare its performance in both normal and abnormal conditions simultaneously and to calculate the positive prediction rate, negative prediction rate and area under the receiver operating characteristic curve (AUC). Second, most of the smartphone-enabled otoscopies in the included studies were performed using devices with iOS. There is little evidence in the existing literature regarding smartphone-enabled otoscopy using devices with other operating systems, let alone comparisons of these systems with traditional otoscopy. Whether different operating systems serve as confounding factors should be clarified in future studies. Third, otoscopy is still an operator-dependent examination. Most of the studies did not have sufficient quantification of the clinical ability of the examiner and sufficient evaluation of the performance of the examination, which may cause potential bias when performing the examination. Fourth, heterogeneity may exist across the included studies. To account for the possible heterogeneity, we performed multiple sensitivity tests and chose a random-effects model to synthesize the results. All of the pooled analyses were consistently significant and did not reverse the initial pooled result. Still, as age is considered a potential confounder, there is an insufficient number of included studies to perform the conclusive meta-regression to determine if age caused heterogeneity. Finally, although the pooled effect estimate showed that smartphone-enabled otoscopy was associated with significantly higher correctness than traditional otoscopy, the results of the TSA showed that at this level of difference, the sample size was insufficient. We look forward to more large-scale clinical studies to obtain comprehensive and solid evidence regarding this issue.

## 5. Conclusions

We compared the correctness between smartphone-enabled otoscopy and traditional otoscopy. While the current evidence showed smartphone-enabled otoscopy was associated with higher correctness in detecting middle ear diseases, the further trial sequential analysis suggested that a larger sample size was required to consolidate the conclusion. In addition to the potential benefit for teaching and training purposes, we expect broader applications of smartphone-enabled otoscopy in the future, considering its availability and affordability.

## Figures and Tables

**Table 1:** Study characteristics.

Study	Study Type	Country	Setting	Ear Condition	Sample (Smartphone/Traditional)	Event Rate (Event/Total)	Mean Age of Examinee (SD)	Level of Examiner	Smartphone-Enabled Otoscopy	Operating System of Smartphone
Kleinman et al. [11] 2021	RCT	USA	ED	AOM	188 (92/96)	Smartphone group: 69/92	6.25 year (3.84)	Resident: 91% (171) Medical student: 9% (17)	CellScope Oto	iOS
						Traditional group: 61/96				
Chan et al. [41] 2019	RCT	USA	ED	AOM	1390 (614/776)	Smartphone group: 123/614	46.79 month (40.68)	Resident	CellScope Oto	iOS
						Traditional group: 146/776				
Mousseau et al. [42] 2018	RCT	Canada	ED	AOM	94 * (188/188)	Smartphone group: 140/188	2.25 year (0.61)	Resident	CellScope Oto	iOS
						Traditional group: 129/188				
Schuter-Bruce et al. [43] 2020	RCT	UK	Simulation	AOM	42 (20/22)	Smartphone group: 15/20	Not applicable	Medical student	TYMPA smartphone otoscope	iOS
						Traditional group: 5/22				
				Perforation	42 (20/22)	Smartphone group: 13/20				
						Traditional group: 3/22				
				VTI	42 (20/22)	Smartphone group: 20/20				
						Traditional group: 20/22				
				Normal	42 (20/22)	Smartphone group: 19/20				
						Traditional group: 11/22				

* The study was designed as a crossover randomized controlled trial. Ninety-four examinees were randomly assigned to undergo smartphone-enabled otoscopy followed by traditional otoscopy or traditional otoscopy followed by smartphone-enabled otoscopy.

## Supplementary Materials

The following are available online at https://www.mdpi.com/article/10.3390/diagnostics12040972/s1: Table S1: Detailed search strategy; Figure S1: Detail of Risk of Bias; Figure S2: Percentage of Risk of Bias.
