# Supplementary material for: Smartphone-Enabled versus Conventional Otoscopy in Detecting Middle Ear Disease: A Meta-Analysis
Source: Diagnostics (Basel). 2022 Apr 13;12(4):972. doi: 10.3390/diagnostics12040972 (PMC9029949; doi:10.3390/diagnostics12040972)

Smartphone-Enabled versus Conventional Otoscopy in Detecting Middle Ear Disease: A Meta-analysis

Chih-Hao Chen, Chii-Yuan Huang, Hsiu-Lien Cheng, Heng-Yu Haley Lin, Yuan-Chia Chu, Chun-Yu Chang, Ying-Hui Lai, Mao-Che Wang, and Yen-Fu Cheng

Table S1. Detailed search strategy

Database	Query
PubMed	(((("Cell Phone"[Mesh]) OR ("Cell Phone/classification"[Mesh] OR "Cell Phone/instrumentation"[Mesh] OR "Cell Phone/statistics and numerical data"[Mesh] OR "Cell Phone/supply and distribution"[Mesh])) OR "Smartphone"[Mesh]) OR ("Smartphone/classification"[Mesh] OR "Smartphone/instrumentation"[Mesh] OR "Smartphone/statistics and numerical data"[Mesh] OR "Smartphone/supply and distribution"[Mesh]) OR "Smartphones"[TIAB] OR "Smart Phones"[TIAB] OR "Smart Phone "[TIAB] OR "Cellular Phone"[TIAB] OR "Cellular Phones"[TIAB] OR "Cellular Telephone"[TIAB] OR "Cellular Telephones"[TIAB] OR "Cell Phones"[TIAB] OR "Portable Cellular Phone"[TIAB] OR "Portable Cellular Phones"[TIAB] OR "Mobile Phone"[TIAB] OR "Mobile Phones"[TIAB] OR "Mobile Telephone"[TIAB] OR "Mobile Telephones"[TIAB] OR "Car Phone"[TIAB] OR "Car Phones"[TIAB] OR "Mobile"[TIAB] OR "iPhone"[TIAB] OR "Smartphones" OR "Smart Phones" OR "Smart Phone " OR "Cellular Phone" OR "Cellular Phones" OR "Cellular Telephone" OR "Cellular Telephones" OR "Cell Phones" OR "Portable Cellular Phone" OR "Portable Cellular Phones" OR "Mobile Phone" OR "Mobile Phones" OR "Mobile Telephone" OR "Mobile Telephones" OR "Car Phone" OR "Car Phones" OR "Mobile" OR "iPhone") AND ("Traditional"[TIAB] OR "Conventional"[TIAB] OR "Standard"[TIAB] OR "Traditional" OR "Conventional" OR "Standard") AND (((("Otoscopy"[Mesh]) OR ("Otoscopy/methods"[Mesh] OR "Otoscopy/standards"[Mesh] OR "Otoscopy/statistics and numerical data"[Mesh] OR "Otoscopy/therapeutic use"[Mesh] OR "Otoscopy/therapy"[Mesh] OR "Otoscopy/veterinary"[Mesh])) OR "Otoscopes"[Mesh]) OR ("Otoscopes/standards"[Mesh] OR "Otoscopes/statistics and numerical data"[Mesh] OR "Otoscopes/therapeutic use"[Mesh]) OR "Otoscopes"[TIAB] OR "Otoscope"[TIAB] OR "Otoscopy"[TIAB] OR "Otoscopies"[TIAB] OR "Otoscopes" OR "Otoscope" OR "Otoscopy" OR "Otoscopies")
Embase	('smartphone'/exp OR 'smart phones' OR 'smart phone' OR 'cellular phone' OR 'cellular phones' OR 'cellular telephone' OR 'cellular telephones' OR 'cell phones' OR 'portable cellular phone' OR 'portable cellular phones' OR 'mobile phones' OR mobile OR 'mobile phone'/exp OR 'mobile telephones' OR 'car phone' OR 'car phones' OR 'iphone'/exp) AND ('otoscope'/exp OR otoscopes OR 'otoscopy'/exp OR otoscopies) AND (traditional OR conventional OR 'standard'/exp) AND ('otoscopy'/exp OR otoscopes OR 'otoscope'/exp OR otoscopies)
Web of Science	TS=(("Smartphones" OR "Smart Phones" OR "Smart Phone " OR "Cellular Phone" OR "Cellular Phones" OR "Cellular Telephone" OR "Cellular Telephones" OR "Cell Phones" OR "Portable Cellular Phone" OR "Portable Cellular Phones" OR "Mobile Phone" OR "Mobile Phones" OR "Mobile Telephone" OR "Mobile Telephones" OR "Car Phone" OR "Car Phones" OR "Mobile" OR "iPhone")AND("Traditional" OR "Conventional" OR "Standard")AND("Otoscopes" OR "Otoscope" OR "Otoscopy" OR "Otoscopies"))
Scopus	(TITLE-ABS-KEY("Smartphones" OR "Smart Phones" OR "Smart Phone " OR "Cellular Phone"

	OR "Cellular Phones" OR "Cellular Telephone" OR "Cellular Telephones" OR "Cell Phones" OR "Portable Cellular Phone" OR "Portable Cellular Phones" OR "Mobile Phone" OR "Mobile Phones" OR "Mobile Telephone" OR "Mobile Telephones" OR "Car Phone" OR "Car Phones" OR "Mobile" OR "iPhone") AND TITLE-ABS-KEY("Traditional" OR "Conventional" OR "Standard") AND TITLE-ABS-KEY("Otoscopes" OR "Otoscope" OR "Otoscopy" OR "Otoscopies"))
Cochrane Library	ID Search<br>#1 MeSH descriptor: [Smartphone] 1 tree(s) exploded<br>#2 MeSH descriptor: [Smartphone] 1 tree(s) exploded and with qualifier(s): [instrumentation - IS]<br>#3 MeSH descriptor: [Smartphone] 1 tree(s) exploded and with qualifier(s): [statistics & numerical data - SN]<br>#4 MeSH descriptor: [Smartphone] 1 tree(s) exploded and with qualifier(s): [classification - CL]<br>#5 MeSH descriptor: [Smartphone] 1 tree(s) exploded and with qualifier(s): [supply & distribution - SD]<br>#6 MeSH descriptor: [Cell Phone] explode all trees<br>#7 MeSH descriptor: [Cell Phone] explode all trees and with qualifier(s): [classification - CL]<br>#8 MeSH descriptor: [Cell Phone] explode all trees and with qualifier(s): [instrumentation - IS]<br>#9 MeSH descriptor: [Cell Phone] explode all trees and with qualifier(s): [statistics & numerical data - SN]<br>#10 MeSH descriptor: [Cell Phone] explode all trees and with qualifier(s): [supply & distribution - SD]<br>#11 Cellular Phone<br>#12 Cellular Phones<br>#13 Cellular Telephone<br>#14 Cellular Telephones<br>#15 Cell Phones<br>#16 Portable Cellular Phone<br>#17 Portable Cellular Phones<br>#18 Mobile Phone<br>#19 Mobile Phones<br>#20 Mobile<br>#21 Mobile Telephone<br>#22 Mobile Telephones<br>#23 Car Phone<br>#24 Car Phones<br>#25 Smartphones<br>#26 Smart Phones

	#27 Smart Phone<br>#28 Smartphone<br>#29 iPhone<br>#30 {OR #1-#28}<br>#31 Traditional<br>#32 Conventional<br>#33 Standard<br>#34 {OR #31-#33}<br>#35 MeSH descriptor: [Otoscopes] explode all trees<br>#36 MeSH descriptor: [Otoscopy] explode all trees<br>#37 MeSH descriptor: [Otoscopy] explode all trees and with qualifier(s): [methods - MT]<br>#38 MeSH descriptor: [Otoscopy] explode all trees and with qualifier(s): [standards - ST]<br>#39 MeSH descriptor: [Otoscopes] explode all trees and with qualifier(s): [standards - ST]<br>#40 Otoscopes<br>#41 Otoscope<br>#42 Otoscopy<br>#43 Otoscopies<br>#44 {OR #35-#43}<br>#45 #30 AND #34 AND #44

Figure S1. Detail of Risk of Bias. The revised Cochrane Risk of Bias Tool 2 was used to evaluate to quality of included studies.

**Studies with intention-to-treat**	Unique ID	Study ID	Experimental	Comparator	Outcome	Weight	Randomization process	Deviations from intended intervention	Missing outcome data	Measurement of the outcome	Selection of the reported result	Overall			
	Kleinman et al, 2021	31	smartphone otoscope	traditional otoscope	Correctness rate	1	+ (green)	? (red)	+ (green)	? (yellow)	+ (green)	! (yellow)		+ (green)	Low risk
	Chan et al, 2019	3	smartphone otoscope	traditional otoscope	Correctness rate	1	+ (green)	? (red)	+ (green)	? (yellow)	+ (green)	! (yellow)		? (yellow)	Some concerns
	Mousseau et al, 2018	6	smartphone otoscope	traditional otoscope	Correctness rate	1	+ (green)	? (red)	+ (green)	? (yellow)	+ (green)	! (yellow)		? (red)	High risk
	Schuter-Bruce et al, 2020	22	smartphone otoscope	traditional otoscope	Correctness rate	1	+ (green)	? (yellow)	+ (green)	? (yellow)	+ (green)	! (yellow)			

Figure S2: Percentage of Risk of Bias. Overall, all the included studies are subject to some concerns of bias. Some concerns on measurement of the out come remain in all of the studies. Some concerns on deviation from inteded interventions remain in twenty five percents of studies. Seventy-five percents of the included studies sustain high risk on on deviation from inteded interventions.

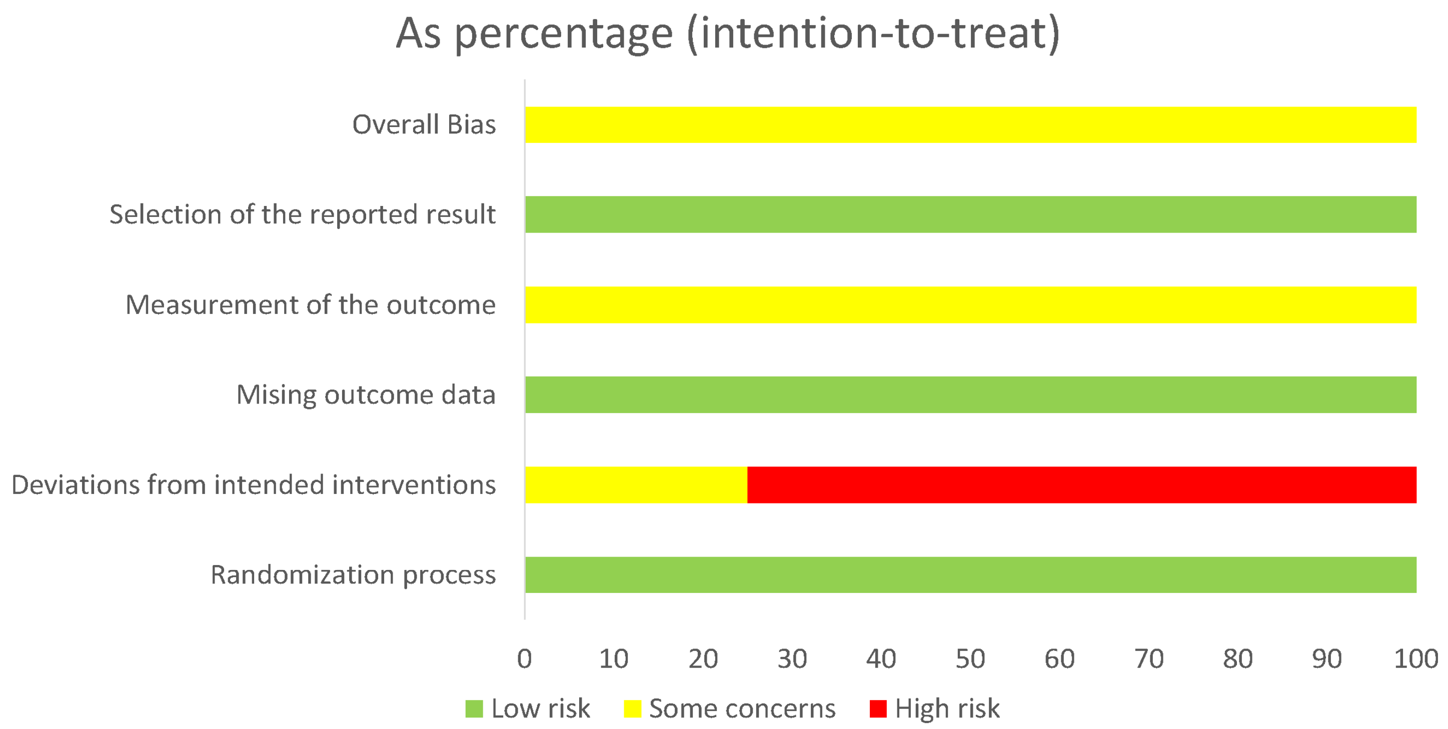

Supplement: Supplementary file 1 [file diagnostics-12-00972-s001.zip › 20220128 Supplementary for MDPI.pdf]
